# The Comparition of the Efficacy of Two Different Probiotics in Rotavirus Gastroenteritis in Children

**DOI:** 10.1155/2012/787240

**Published:** 2012-06-19

**Authors:** Özlem Erdoğan, Bilge Tanyeri, Emel Torun, Erdem Gönüllü, Hüseyin Arslan, Ufuk Erenberk, Faruk Öktem

**Affiliations:** ^1^Department of Pediatrics, Medical Faculty, Bezmialem Vakif University, Adnan Menderes Bulvarı, P.K.: 34093 Fatih, Istanbul, Turkey; ^2^Department of Neonatal Intensive Care Unit, Medical Faculty, Bezmialem Vakif University, Istanbul, Turkey

## Abstract

*Objectives*. The aim of the study is to compare the clinical effectiveness of the probiotics—*Saccharomyces boulardii* and *Bifidobacterium lactis*—in children who had been diagnosed with rotavirus gastroenteritis. *Materials and methods*. Seventy five patients aged between 5 months–5 years diagnosed as rotavirus gastroenteritis were included in the study. The patients diagnosed as rotavirus gastroenteritis by latex agglutination test in stool were divided into 3 groups of twenty-five patients each: First group was given oral rehydration therapy and rapid refeeding with a normal diet with *Saccharomyces boulardii* (*spp. I-745*), second group was given oral rehydration therapy and rapid refeeding with a normal diet with *Bifidobacterium lactis* (*spp. B94, culture number:N*°118529) and third group received only oral rehydration therapy and rapid refeeding with a normal diet. *Results*. The duration of diarrhea was shorter in the group given oral rehydration therapy and rapid refeeding with a normal diet with *Bifidobacterium lactis* and *Saccharomyces boulardii* than the group given only oral rehydration therapy and rapid refeeding with a normal diet. *Conclusion*. *Bifidobacterium lactis* has a complemental role in the treatment of rotavirus gatroenteritis and other probiotics may also have a beneficial effect in rotavirus gastroenteritis compared with the therapy included only oral rehydration therapy and rapid refeeding with a normal diet.

## 1. Introduction

Acute infectious gastroenteritis is one of the most common infectious diseases of children resulting in high mortality rates in developing countries and high morbidity rates worldwide. Rotavirus is the most common agent causing acute diarrhea in infants and in children, resulting in 25 million physician visits and 2 million hospitalizations every year [1]. Gastroenteritis results in approximately 600.000 deaths annually [2].

Probiotics are living microorganisms which, when administered in sufficient quantities, have a beneficial effect on the host. They are largely anaerobic organisms and prevent pathogenic microorganisms from growing in the human gut. They have resistance to gastric and bile acidity and could adhere to mucus and/or human epithelial cells and show antimicrobial activity against potentially pathogenic bacteria. Probiotics have the ability to reduce pathogen adhesion to surfaces [3]. Derived from food sources such as cultured milk products, these microorganisms include lactic acid bacteria (e.g., *Lactobacillus* and *Bifidobacterium*), a nonpathogenic strain of *Escherichia coli* (e.g., *E. coli *Nissle, 1917), *Clostridium butyricum*, *Streptococcus salivarius* and *Saccharomyces boulardii *(a non pathogenic strain of yeast). Probiotics are recommended to use in childhood diarrhea, childhood allergies, antibiotic associated diarrheas, inflamatory bowel disease and irritable bowel syndrome [4]. Probiotics are also used in preventation and therapy of childhood diarrhea, however, therapy has not been standardized and the most effective and safe organism had not been identified yet [5–7]. 

Different studies about probiotic therapies for acute diarrhea in children showed beneficial effects in rotavirus gastroenteritis. This study aims to determine more accurately which of the probiotics (i.e., *Saccharomyces boulardii* and *Bifidobacterium lactis*) in combination with oral rehydration therapy and rapid refeeding with a normal diet will provide optimal effectiveness in the treatment of acute rotavirus gastroenteritis.

## 2. Subjects and Methods

The study was a prospective, randomized one that included seventy-five children (38 female, 37 male) aged between 5 months and 5 years old. The patients admitted to the Bezmialem Hospital Pediatric Emergency Department with 3 or more times of watery diarrhea per day in the last 48 hours and diagnosed as rotavirus gastroenteritis. The study was conducted between October, 2009 and May, 2010.

Each patient's medical history (including rotavirus vaccination), demographics, degree of dehydration, and the symptoms associated with gastroenteritis (frequency of diarrhea, stool characteristics consistency, and vomiting) were recorded.

Clinical assessment of dehydration was estimated as mild dehydration (<5%, normal or increased pulse, decreased urine output, thirsty, and normal physical findings), moderate dehydration (5–10%, tachycardia, little or no urine output, irritability, lethargy, sunken eyes and fontanel, decreased tears, dry mucus membrane, mild delay in elasticity, delayed capillary refill), and severe dehydration (>10%, rapid or weak or absent peripheral pulses, decreased blood pressure, no urinary output, very sunken eyes and fontanel, no tears, parched mucous membrane, delayed elasticity, very delayed capillary refill, depressed consciousness), and the patients with severe dehydration had been excluded from the study. The patients estimated as moderate dehydration with no oral tolerance had needed to be hospitalized.

Stool specimens were obtained as early as possible after admittance to the hospital and were examined for stool cultures, microscopic examination for bacterial pathogens and parasites and rotavirus antigen by latex agglutination tests (SD BIOLINE rota/adeno rapid kit, SD Diagnostics INC, Korea). The patients whose stool tests and microscopic examination were positive for bacterial pathogens were excluded from the study.

The patients whose stool tested positive by the rotavirus antigen test were divided into 3 groups of twenty-five patients each. First group was given oral rehydration therapy and rapid refeeding with a normal diet with 282.5 mg/day *Saccharomyces boulardii* (spp*. I-745*, Reflor sache, Biocodex). Second group was given oral rehydration therapy and rapid refeeding with a normal diet with 30 mg/day *Bifidobacterium lactis* (spp. B94, culture number: *N*°118529, Maflor sache, Mamsel). Third group received only oral rehydration therapy and rapid refeeding with a normal diet. 

Patients were followed up in the hospital until oral hydration was possible and postdischarged followup, were done by telephone to elicit frequency of diarrhea, stool characteristics and consistency, and episodes of vomitting per day.

The study was approved by the local ethical committee and informed written consent was obtained from parents. Statistical calculations were made by the SPSS 19 programme for Windows. Categorical data were evaluated using the Chi-squared test. Repeated ANOVA test was used in calculating parameters of more than two variables between different groups, and *P* < 0.05 was accepted as statistically significant.

## 3. Results

In total, 62.7% of the cases were presented in winter, 30.7% in spring, and 6.7% were in autumn. The mean age of all patients was 20.9 ± 12.8 months. The mean age of the group treated with oral rehydration therapy and rapid refeeding with a normal diet with *Saccharomyces boulardii* was 21.6 ± 11.5 months; the mean age of group treated with oral rehydration therapy and rapid refeeding with a normal diet with *Bifidobacterium lactis* was 22.1 ± 14 months; the mean age of group treated with oral rehydration therapy and rapid refeeding with a normal diet was 19.1 ± 13.3 months. Gender distribution between the three groups was similar, with a total of 49.3% males and 50.7% females that was not statistically significant (*P* > 0.05).

Dehydration scores were evaluated due to the physical findings in the first assessment of the patients and in all groups; dehydration scores were not statistically significant (Table 1).

The mean duration time of diarrhea in all groups was 5.9 ± 2 days: 6.6 ± 1.7 days for the first group, 4.1 ± 1.3 days for the second group, and 7.0 ± 1.6 days for the third group (Table 2). The mean of the duration time of diarrhea in the second group supplemented with *Bifidobacterium lactis* was significantly shorter than the mean duration times of the first and third groups (*P* < 0.001) (Table 2).

Throughout the therapy, the patients were followed up daily. In total, 44% of them required hospitalization for intravenous hydration therapy, specially 48% of the patients in the group treated with oral rehydration therapy and rapid refeeding with a normal diet with *Saccharomyces boulardii*, 40% in the group treated with oral rehydration therapy and rapid refeeding with a normal diet with *Bifidobacterium lactis,* and 44% in the group treated with oral rehydration therapy and rapid refeeding with a normal diet.

**Figure 1:**
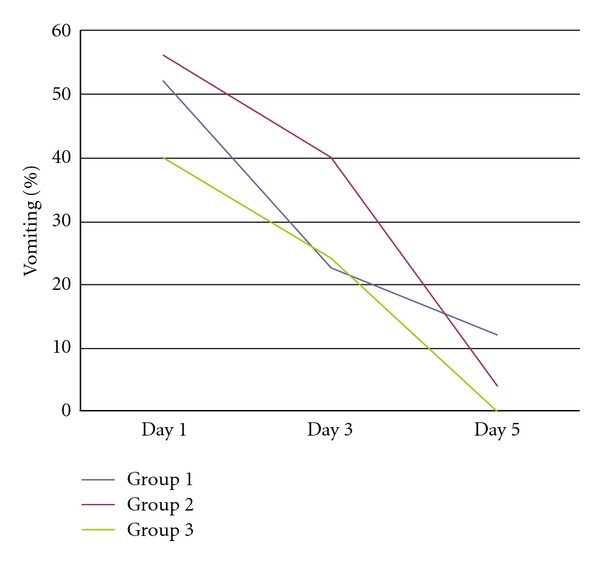
Vomiting rates in all groups.

Vomiting was prominent in 49.3% of all the patients. Vomiting was associated with watery diarrhea in 52% of the patients in group 1; 56% of the patients in group 2, 40% of the patients in group 3 (Table 3). Vomiting rates had no significant difference in all groups Vomiting rates were higher in the first 3 days and decreased, respectively, in followup (Figure 1).

Of the 75 patients, 93.3% had not been vaccinated with rotavirus vaccine. In other words, our study patients had, in total, a 6.7% vaccination rate: 0% in group 1, 16% in group 2, and 4% in group 3.

## 4. Discussion

Acute rotavirus diarrhea remains a major problem in infants and in children resulting in substantial morbidity, mortality, and financial cost. Rotavirus is the agent most commonly causing diarrhea in children between six months and two years of age [8, 9], with incidence and severity diminishing after age five [10, 11]. Our study was consistent with the epidemiological studies, those in the literature in that most of the patients were younger than two years old (with the mean of 20.9 months). Gender distrbuition of the three groups were similiar.

Rotavirus infection has seasonal characteristics with peak incidence typically in winter [10–13]. In our study almost two-thirds of the cases (62.7%) were presented in winter, 30.7% in spring, and only 6.7% were in autumn. 

The priority in gastroenteritis therapy is to replace the fluid loss and reduce the severity and duration of diarrhea. Different supportive measures include fluid repletion, antiemetic agents, zinc, and probiotics. Probiotics are commonly used in viral diarrhea in order to suppress the growth or epithelial invasion of pathogenic bacteria in the human gut, improve the intestinal barrier function, modulate the immune system of the intestine, and mediate analgesic functions [14, 15].

Specific probiotics are commonly used in acute rotavirus diarrhea, but limited data were found as to their therapeutic efficacy. Our study compared the effects of the probiotics *Saccharomyces boulardii* and *Bifidobacterium lactis* in rotavirus gastroenteritis. The mean duration time of diarrhea in the group given oral rehydration therapy and rapid refeeding with a normal diet with *Bifidobacterium lactis* was significantly shorter than the first and third groups. Vomiting was associated with watery diarrhea in all groups but less in the third group treated with oral rehydration therapy and rapid refeeding with a normal diet.

Several studies have evaluated probiotics in the treatment of infectious diarrhea in infants and in children with heterogenous results. The earliest study conducted by Isolauri et al. aimed to determine the effect of a human Lactobacillus strain (*Lactobacillus casei* sp. strain GG) on recovery from acute diarrhea (82% rotavirus) on well-nourished children between 4 and 45 months of age. The study showed that *Lactobacillus GG* in the form of fermented milk or freeze-dried powder is effective in shortening the course of acute diarrhea [16]. A similar study about the effect of *Lactobacillus GG* on the duration of both viral and bacterial diarrhea concluded that *Lactobacillus GG* significantly shortened the duration of rotavirus diarrhea but not diarrhea with confirmed bacterial aetiology [17]. Children receiving the multiple species had shorter duration time of diarrhea and fewer episodes of vomiting in the study that compare, oral rehydration therapy plus placebo with oral rehydratation therapy plus *Saccharomyces boulardii* and oral rehydratation therapy plus compound probiotics (containing *Lactobacillus acidophilus*, *Lactobacillus rhamnosus*, *Bifidobacterium longum,* and *Saccharomyces boulardii*) conducted by Grandy et al. [18]. A similiar study conducted by Canani et al. revealed that the duration of diarrhea was significantly lower in children who received *Lactobacilus GG* and bacterial mix than those patients who received oral rehydration alone [19]. A meta-analysis about probiotic therapy for acute diarrhea in children was associated with a significant reduction in duration of diarrhea especially in rotavirus gastroenteritis [20]. Another meta-analysis, on the effect of several strains of lactobacilli in children with acute infectious diarrhea, revealed that probiotics reduced the duration and frequency of diarrhea [21]. The study that focused on clinical trials performed on acute-onset, infectious diarrhea showed a significant benefit and moderate clinical benefit on a few, well-identified probiotic strains—mostly *Lactobacillus GG* and *Saccharomyces boulardii*—in the treatment of acute watery diarrhea and particularly those due to rotavirus [22]. The studies using several different probiotic preparations in adults and children concurred that probiotics reduced the overall risk of having diarrhea causes, as well as hastened recovery from acute diarrhea in children [23]. 

Prevention against rotavirus infection included improvements in water supply, hygiene, sanitation of the food, and vaccination. Vaccination against rotavirus could, at minimal expense, reduce the burden of the disease worldwide, particularly when compared with the costs of outpatient visits and hospitalization [24–26]. Although the World Health Organization has given priority to rotavirus vaccination, in Turkey few infants have the opportunity to receive one because it is not included in the national immunization program. In our study, 93.3% of the patients had never received a rotavirus vaccination that reflecting the rotavirus vaccination rates in lower socioeconomical sections of Turkey.

Compared with oral rehydration therapy alone, specific probiotics significantly reduced the duration of hospitalization and duration of diarrhea in a strain-specific manner. *Bifidobacterium lactis* has a complemental role in the treatment of rotavirus gatroenteritis and other probiotics may also have a beneficial effect in rotavirus gastroenteritis, compared with the therapy included only age appropriate diarrhea diet. The effects of combining probiotics were similiar to administration of single probiotics. Effective immunization aganist rotavirus might reduce the morbidity, mortality and cost of the disease; however, at present no universal drug and therapeutic efficacy have been shown for rotavirus gastroenteritis. The therapeutic strategies need to be assessed in different settings, and pharmacoeconomic and nutrition economic benefits should be analyzed on a country-specific basis.

## Figures and Tables

**Table 1:** Initial demographical and clinical features of the patients.

	Group 1^∗^ (*n* = 25)	Group 2^∗∗^ (*n* = 25)	Group 3^∗∗∗^ (*n* = 25)	
Age (months)	21.6 ± 11.5	22.1 ± 14	19.1 ± 13.3	*F* = 0.38
				*P* = 0.68
Gender				
Male	11 (44%)	12 (48%)	14 (56%)	*χ* ^2^ = 0.75
Female	14 (56%)	13 (52%)	11 (44%)	*P* = 0.69
Dehydration score				
<%5	12 (48%)	14 (44%)	13 (52%)	*χ* ^2^ = 0.32
% 5–10	13 (52%)	11 (56%)	12 (48%)	*P* = 0.85
Hospitalization rate	12 (48%)	10 (40%)	11 (44%)	*χ* ^2^ = 0.32
				*P* = 0.85

*n*: number of subjects, *F*: one-way ANOVA test statistics.

^∗^Group 1: treated with oral rehydration therapy and rapid refeeding with a normal diet with *S. boulardii.*

^∗∗^Group 2: treated with oral rehydration therapy and rapid refeeding with a normal diet with *B. lactis.*

^∗∗∗^Group 3: treated with oral rehydration therapy and rapid refeeding with a normal diet.

**Table 2:** The duration time (day) of diarrhea in all groups.

Groups	*n*	The mean duration time of diarrhea (day)	*P*
1^∗^	25	6.6 ± 1.7	*F* = 25.94 *P* < 0.001
2^∗∗^	25	4.1 ± 1.3	
3^∗∗∗^	25	7.0 ± 1.6	
Total	75	5.9 ± 2	

*n*: number of subjects, *F*: post hoc Tukey's HSD 2 versus 13 (*P* < 0.05).

^∗^Group 1: treated with oral rehydration therapy and rapid refeeding with a normal diet with *S*. *boulardii*.

^∗∗^Group 2: treated with oral rehydration therapy and rapid refeeding with a normal diet with *B*.  *lactis*.

^∗∗∗^Group 3: treated with oral rehydration therapy and rapid refeeding with a normal diet.

**Table 3:** Rate of vomiting episodes per day in followup in all groups.

Follow-up day		1st day	3rd day	5th day
Groups				
1^∗^	*n*	13	9	3
	%	52%	22.5%	12%
2^∗∗^	*n*	14	10	1
	%	56,0%	40%	4%
3^∗∗∗^	*n*	10	6	—
	%	40,0%	24%	0%

	*P*	0,85	0,46	0,157

Total	*n*	37	25	4
	%	49.3%	33.3%	16%

^∗^Group 1: treated with oral rehydration therapy and rapid refeeding with a normal diet with *S*.  *boulardii.*

^∗∗^Group 2: treated with oral rehydration therapy and rapid refeeding with a normal diet with *B*.  *lactis.*

^∗∗∗^Group 3: treated with oral rehydration therapy and rapid refeeding with a normal diet.
